# Evaluation of the BCL Clinicon Chemcode system

**DOI:** 10.1155/S1463924681000096

**Published:** 1981

**Authors:** A. D. Di Michiel, R. J. Wood, H. G. Worth, D. G. Williams

**Affiliations:** 1 Department of Clinical Chemistry Northwick Park Hospital and Clinical Research Centre Harrow Middlesex 141 3UJ UK; 2 The Canberra College of Advanced Education ACT Canberra Australia; 3 Courtauld Institute of Biochemistry Middlesex Hospital Medical School London W1 UK


### Evaluation of the BCL Clinicon Chemcode system

**A.D.Di Michiel,* R.J. Wood,** H.G. Worth and D.G. Williams**

*Department of Clinical Chemistry, Northwick Park Hospital and Clinical Research Centre, Harrow, Middlesex, HA1 3UJ.*

### Introduction

The Clinicon Chemcode operates as a microprocessor-controlled photometric system and with a suitable reagent programme will perform measurements for the batch analysis of enzymes and substrates. It is intended to perform a wide range of routine clinical chemistry assays in a small laboratory. In a larger laboratory it provides a versatile emergency system and a back-up for existing instrumentation. A detailed description of the complete system will not be given since this is available from the manufacturer, Biodynamics Inc, USA, a divison of Boehringer Mannheim Corp. In the UK, the instrument is marketed by Boehringer Corp. Ltd, Lewes, East Sussex.

The operator pre-programmes the instrument with the following information for each of the twenty different test selections which are possible: test identity, wavelength, mode of analysis (either full kinetic, fixed point kinetic or endpoint). Addition parameters are required depending on the mode selected, viz, for the endpoint mode the blank value and factor, for the full kinetic mode the factor and linearity tolerance and for the fixed point kinetic mode the first and second measuring times as well as the factor. Samples are prepared for analysis by dilution, mixing with reagent and incubating as directed by the instructions supplied with the reagent programme and method guide for the relevant test. The sample is then introduced into the analyser either by manually placing a cuvette containing the sample into the cuvette holder, or by allowing the microprocessor to control the aspiration of sample into a flow cell. The analyser then performs photometric measurements and processes the results according to the pre-programmed mode and information stored in the microprocessor memory. The final analytical result together with the relevant information is printed out on a dot matrix 5 digit alphanumeric printer.

The evaluation was carried out using the manual method of operation, without the flow cell, to determine the instrument's analytical performance in all three modes of operation, its suitability as back-up for an automated clinical chemistry analyser and as a 'stat' instrument, ie for emergency, out-of-normal-hours work. The study was divided into four parts, a functional evaluation in relation to instrumental factors which would affect analytical performance, a procedural evaluation to ascertain the precision of sample preparation prior to analysis, an analytical evaluation and an electrical evaluation.

### Functional evaluation

### Lamp stability

The optical system operates on a split beam principle. Light from a 45 W mercury lamp is filtered by one of a selection of five interference filters of 365, 405, 436, 546 and 578 nm with specified half band width of 0.5 to 3 nm. Filter selection is pre-programmed. The main and reference beams (ratio 90:10) have separate detectors, the reference beam monitoring the optical system, if the detected intensity falls below a pre-determined level, the READY indicator lamp goes out and the instrument becomes non-operational.

The mercury lamp stability was studied by measuring the absorbance of an empty glass cuvette (against an air blank) over a 2–3 hour period. The observed absorbance drift was less than ±0.005 absorbance units at each of the five wavelengths.

### Cuvette volume

The minimum cuvette volume which could be used in the manual method without the use of the flow-through system was determined to test the suitability of the instrument for micro-analysis. The instrument was set up in the endpoint mode at 365 nm with the blank set to zero and the factor at unity. Absorbance measurements were made after potassium dichromate solution (50 mg/l) was added to the cuvette in 100 $\mu$l portions. The volume (700 $\mu$l) required to give a constant absorbance was observed, ie the minimum necessary for satisfactory operation. Since most of the common analyses could be carried out on 20 $\mu$l of serum, the instrument is suitable for paediatric work.

### Effect of stray light

The instrument does not employ a cut-off system operating when the cuvette holder compartment is opened. Since there is a significant difference between the absorbance readings when the lid is open or closed it is most important that the cuvette compartment is closed whenever absorbance readings are taken. Slight changes in absorbance (±0.003) were noted when a 60 W light bulb was placed near to the sides of the closed compartment lid. Under normal laboratory lighting however stray light entering in this way should not be a problem.

### Accuracy of filters

Solutions of chromogens whose wavelengths for maximum absorbance ($\lambda$ max) were the same as, or near to, the quoted wavelengths of the analyser filters were prepared at three different concentrations. Absorbances obtained at the filter wavelength were compared with measurements obtained on the same samples, at the same wavelengths, using a Pye Unicam SP 1800 spectrophotometer. The wavelength and absorbance accuracy of the SP 1800 had previously been checked as satisfactory.

By this method an indirect check of filter accuracy was carried out, a direct check was not possible because the filters could not easily be removed from the instrument. Data presented in Table 1 show a good agreement except at 365 nm where significant differences were observed, the

**On sabbatical leave from The Canberra College of Advanced Education, Canberra, ACT, Australia.*

***Present address: Courtauld Institute of Biochemistry, Middlesex Hospital Medical School, London W1.*

Chemcode giving a lower absorbance. The greatest divergence often occurs in the mid range of absorbance. Further comment is given under 'Conclusions'.

### Linearity of response

Solutions of the chromogens listed in Table 1 were prepared at seven different concentrations to give an absorbance range 0.100 to 1.000. Absorbance and concentration were compared and in all cases the responses were linear.

### Cuvette holder temperature control

Temperature control of the incubation and measuring stages of the analysis was achieved by pumping water from a thermostatted water bath. The pump/heater control unit used in this evaluation was a Precitherm PFV (Labova, Mannhein, Germany) immersion thermostat accurate to ±0.1°C. One cuvette containing water was placed in the water bath with the control preset at 37°C and another in the photometer cuvette holder. The temperature of the water inside the cuvettes was measured using a calibrated thermistor device; overall temperature variation after 10 min was less than ±0.2°C at 37°C.

**Table 1. Assessment of filter accuracy**

| Filter (nm) | Chromogen (λ max) | Absorbance | |
|---|---|---|---|
| | | Chemcode analyser | SP 1800 |
| 365 | Pottassium dichromate (358) | 0.060 | 0.075 |
| | | 0.576 | 0.738 |
| | | 0.940 | 1.08 |
| 405 | Potassium ferricyanide (420) | 0.069 | 0.076 |
| | | 0.440 | 0.501 |
| | | 0.878 | 0.914 |
| 436 | Methyl red (alkaline) (435) | 0.075 | 0.086 |
| | | 0.658 | 0.710 |
| | | 0.935 | 0.972 |
| 546 | Thymol blue (acidic) (547) | 0.049 | 0.042 |
| | | 0.499 | 0.536 |
| | | 0.952 | 0.962 |
| 578 | Phenolphthalein (alkaline) (550) | 0.047 | 0.047 |
| | | 0.511 | 0.512 |
| | | 0.871 | 0.910 |

### Accuracy of reaction time mechanism

The intervals for the fixed point kinetic (FP) and kinetic (KIN) modes were determined by measuring with a stop watch the time lapse in the printer response between initial and final printout of absorbance values. Checks were made in the FP mode in the range 15 to 60 sec and in the KIN mode at the minimum reaction interval (40 sec). In all cases the true time interval was measured to within ±0.1 sec which is within the precision of measurement with the stop watch.

### Procedural evaluation

Since all solutions for absorbance measurements were prepared in plastic cuvettes by sampling, dilution and dispensing using the BCL Clinicon 2075 diluter, both the optical variation between the cuvettes and the precision of the diluter's dispensing were evaluated.

### Cuvette variation

Two experiments were performed to assess the precision in measuring (a) the absorbance of a single cuvette a number of times, and (b) the variation in the absorbance of a number of cuvettes. In both experiments a mixed indicator alkaline solution (phenolphthalein and methyl red, both 8 mg/l concentration) was used.

In the first experiment, a single cuvette containing the mixed indicator solution was removed from the 37°C water bath and after wiping the outside quickly with tissue paper, positioned in the thermostatted (37°C) cuvette holder in the photometer; the absorbance was measured immediately. This procedure was repeated ten times at each wavelength. In the second experiment one measurement was made on each of 50 cuvettes at each wavelength. The results presented in Table 2 indicate that the variation may be wavelength dependent, and while the same cuvette may be repositioned reproducibly in the instrument there is significant variation between cuvettes.

### Diluter precision

Portions of pooled human serum, spiked with iodine–125 labelled albumin were sampled and dispensed directly into plastic sample tubes with 1.0 ml portions of distilled water using the BCL Clinicon diluter and then counted on a Nuclear Enterprise NE 1600 gamma ray sample counter. The

**Table 2. Precision data for within-batch and between-batch variation in cuvettes**

| Filter | Number of cuvettes | Number of readings per cuvette | Absorbance means | SD | Coefficient of variation |
|---|---|---|---|---|---|
| 365 | 1 | 10 | 0.497 | 0.002 | 0.39 |
| 365 | 50 | 1 | 0.497 | 0.012 | 2.55 |
| 405 | 1 | 10 | 0.500 | 0.004 | 0.82 |
| 405 | 50 | 1 | 0.500 | 0.005 | 0.95 |
| 436 | 1 | 10 | 0.498 | 0.002 | 0.39 |
| 436 | 50 | 1 | 0.508 | 0.007 | 1.35 |
| 546 | 1 | 10 | 0.670 | 0.002 | 0.30 |
| 546 | 50 | 1 | 0.672 | 0.012 | 1.84 |
| 578 | 1 | 9 | 0.257 | 0.002 | 0.70 |
| 578 | 50 | 1 | 0.262 | 0.006 | 2.33 |

**Table 3. BCL Clinicon 2075 diluter dispensing precision data**

| Serum volume sampled (μl) | Water dispensing volume (ml) | Number of samples | Counting time (sec) | Counts | | |
|---|---|---|---|---|---|---|
| | | | | Mean | SD | % CV |
| 10 | 1.0 | 19 | 100 | 10663 | 257 | 2.41 |
| 20 | 1.0 | 20 | 50 | 10646 | 259 | 2.44 |
| 50 | 1.0 | 20 | 20 | 10738 | 168 | 1.55 |
| 100 | 1.0 | 20 | 10 | 10372 | 124 | 1;20 |

The background count was less than 1 cps, and the precision at 10,000 counts is 1%.

results of this experiment are presented in Table 3. The variation at lower sample volumes is comparable with the optical variation between cuvettes.

### Analytical evaluation

The analytical performance of the instrument was determined by carrying out analyses on specimens received for routine investigation. The results were compared with those obtained using the usual laboratory methods, also quality control specimens were repeatedly analysed to measure between-batch variation. Both kinetic and endpoint modes were examined ensuring that all filters (except the 436 for which no routine chemistry tests were available) were used for at least one type of analysis. The analyses selected were glucose, calcium and urea for the endpoint mode, and alkaline phosphatase, aspartate aminotransferase and γ-glutamyltransferase for the kinetic mode, γ-glutamyltransferase was also investigated in the fixed point kinetic mode. A minimum number of 100 specimens was analysed for all modes and specimens were selected for each analyte so that at least 10% were outside the reference range. The data were analysed (with an ICL 1903A computer) to obtain standard deviation (SD) and coefficient of variation (CV). Methods were compared with standard routine laboratory procedures. The data were analysed using the paired test and by determining the regression equation $y = a + bx$ ($y$ = Chemcode data, $x$ = routine method result) and the correlation coefficient (r).

### Endpoint mode (EP)

Details of the methods used on the instrument are shown in Table 4. In each case the same principle was used in the comparison method except for urea where a Berthelot type method [4] was used. The blank and factor parameters were obtained from duplicate absorption measurements of the reagents blank and an aqueous standard solution respectively.

The linearity of each method was checked by measuring the absorbance of aqueous solutions of known concentration over a reasonable working range and was found to be acceptable.

The statistical analysis of the data is shown in Table 5.

**Table 4. Methodological details of the analyses**

| Analysis | Chemcode | | | | Comparison | |
|---|---|---|---|---|---|---|
| | Mode | Sample volume (ul) | Final volume (ml) | Filter | Instrument | Wavelength (nm) |
| Glucose<br>Glucose oxidase (1) | EP | 10 | 1.01 | 546 | Vickers D300 | 505 |
| Urea<br>Enzymatic (Boehringer Diagnostics Urea UV System) | EP | 5 | 1.005 | 365 | Vickers M300 | 560 |
| Calcium<br>Cresolpthalein complexone (2) | EP | 20 | 1.22 | 578 | Vickers M300 | 595 |
| Aspartate aminotransferase<br>Boehringer Diagnostics 'UV method' | KIN | 150 | 1.25 | 365 | Coulter Kem-O-Mat | 340 |
| γ-Glutamyl transferase<br>Boehringer Diagnostics γ-GT New | KIN & FP | 100 | 1.20 | 405 | Coulter Kem-O-Mat | 405 |
| Alkaline phosphatase<br>p-nitrophenol phoshate (3) | KIN | 10 | 1.11 | 405 | BCL Clinicon Rate Reaction Analyser | 405 |

**Table 5. Correlation of data from 100 patient specimens**

| Test | Mode | Intercept (a) | Slope (b) | Correlation coefficient (r) | SD of differences | Paired t test | |
|---|---|---|---|---|---|---|---|
| | | | | | | t | p |
| Glucose | EP | 0.01 | 1.03 | 0.998 | 0.04 | 7.16 | $<0.001$ |
| Urea | EP | 0.39 | 0.88 | 0.965 | 0.13 | 2.98 | $0.01>p>0.001$ |
| Calcium | EP | 0.17 | 1.13 | 0.827 | 0.01 | 10.48 | $<0.001$ |
| Alkaline phosphatase | KIN | 15.82 | 0.96 | 0.983 | 2.94 | 2.24 | $0.01>p>0.001$ |
| Aspartate transaminase | KIN | 7.93 | 0.86 | 0.989 | 1.64 | 0.42 | $>0.5$ |
| γ-Glutamyl transferase | KIN | 0.29 | 1.05 | 0.995 | 1.09 | 3.11 | $0.01>p>0.001$ |
| γ-Glutamyl transferase | FP | 4.05 | 0.94 | 0.990 | 1.43 | 0.15 | $>0.5$ |

All results compare well and although in each case the paired t test shows that the analyses are significantly different, this is probably due to the small differences between the means, ie glucose 0.27, urea 0.40, and calcium 0.13 mmol/l.

The within-batch and between-batch precision for each of the three analytes was determined at two concentrations by repeated analysis (10 times) of two control specimens; the results are presented in Table 6. The precision for glucose was within the range acceptable for a manual micro-method. The between-batch precision for calcium was not as good as expected, possibly because of repeated use of the disposable cuvettes. This is a very sensitive assay and therefore new plastic cuvettes should be used at all times. There was difficulty in obtaining good within-batch precision with the urea analyses because the small sample volume (5 μl) required, cannot be dispensed on the BCL Clinicon diluter. A 5 μl Eppendorf pipette proved unsatisfactory, and so a Microlab-P programmable microprocessor-controlled hand pipette containing a 100 μl Hamilton syringe was used. Although this improved the precision, the within-batch data were still unacceptable and therefore the between-batch precision was not determined. This is a methodological and not an instrumental problem.

**Kinetic mode (KIN)**

The analytes and details of the methods used in the KIN mode are shown in Table 4. The linearity tolerance was set to 10%, and in almost all cases where a non-linear result was initially obtained, reanalysis of the same reaction mixture gave a linear result. The extra pre-incubation period allowed the non-specific reactions to go to completion. Only linear results were used as data for the evaluation. The statistical analysis of the data is shown in Table 5.

In each case the Chemcode and the comparison method results correlated well, although except for aspartate aminotransferase, the paired t test gave statistically significantly different results. The differences between the means for the two methods were alkaline phosphatase, 6.6. IU/l, aspartate transaminase, 0.7 IU/l and γ-glutamyl transferase, 3.4 IU/l. Both within-batch and between-batch precision were determined in the same way as for the endpoint analyses; the precision details shown in Table 6 are within acceptable limits.

**Fixed point kinetic mode (FP)**

Specimens for γ-glutamyl transpeptidase were assayed using the same clinical method and specimens as for the KIN mode. Fixed point time intervals T1 and T2 were set at 15 and 75 sec respectively, and the factor was taken directly from the kit's method specifications. The statistical analysis of the date (Table 5) gives a correlation coefficient which is almost the same as that obtained using the KIN mode. Because there is no linearity check, some measurements accepted in this mode may be outside the linear requirements laid down for the KIN mode. Preferably, therefore, kinetic assays should be carried out in the KIN rather than FP mode.

### Electrical evaluation

A general electrical inspection and evaluation of both photometer and printer was carried out with special attention being given to electrical safety using the recommendations outlined in "Electrical Safety Code for Hospital Laboratory Equipment" [5]. The figures quoted in parenthesis in this section indicate the relevant paragraphs in the Safety Code. Both the photometer and printer modules were well constructed, robust and easy to handle. The wiring of both was generally good and the microprocessor and signal handling boards were of a standard slot-in variety affording easy access. However, the printer output bus from the photometer bus cable comes very close to the sharp edge of the power supply screen which in time could cut through the insulation (para 90). The colours of the mains supply cords are also incorrect (para 160) as they are white for live, black for neutral and green for earth. The accessibility of the photometer was adequate with the indicator bulbs under the keyboard buttons being easily replaced. The lamp could also be replaced with care without removing the outer casing. However, the power supply could be rather difficult to test or repair as it is inaccessible in places and the lack of an air filter in the cooling system could lead to components becoming dusty and malfunctioning.

The siting of the on/off switch on the photometer module is unsatisfactory as it is in an inaccessible position at the rear of the instrument, and directly below an inadequately guarded cooling fan which can be touched with the fingers. The on/off switches on both modules contravene several paragraphs in the Safety Code (paras 169-171) because there

**Table 6. Precision studies with control sera**

| | In-batch precision | | | | Between-batch precision | | | |
|---|---|---|---|---|---|---|---|---|
| | n | Mean | SD | CV | n | Mean | SD | CV |
| Glucose (mmol/l) | 10 | 5.36 | 0.19 | 3.5 | 10 | 5.04 | 0.24 | 4.9 |
| | 10 | 11.23 | 0.46 | 4.1 | 10 | 10.91 | 0.61 | 5.6 |
| Calcium (mmol/l) | 10 | 2.50 | 0.04 | 1.7 | 10 | 2.73 | 0.30 | 10.8 |
| | 10 | 2.97 | 0.04 | 1.3 | 10 | 3.51 | 0.43 | 12.2 |
| Urea (mmol/l) | 10 | 9.86 | 2.47 | 25.1* | | | | |
| | 10 | 7.5 | 0.8 | 10.2** | | | | |
| Alkaline phosphatase (IU/l) | 10 | 159 | 9 | 5.7 | 10 | 123 | 17 | 13.7 |
| | 10 | 348 | 15 | 4.3 | 10 | 309 | 25 | 8.2 |
| γ-Glutamyl transferase (IU/l) | 10 | 22 | 2 | 7.2 | 10 | 16 | 4 | 25.4 |
| | 10 | 34 | 2 | 6.3 | 10 | 30 | 3 | 9.2 |
| Aspartate transaminase (IU/l) | 10 | 67 | 3 | 3.9 | 10 | 68 | 2 | 3.6 |
| | 10 | 164 | 6 | 3.8 | 10 | 168 | 6 | 3.8 |

* *Using 5 μl Eppendorf pipette*

** *Using 5 μl programmable Hamilton syringe*

are no visual warnings (ie lamp) to show that the current is on, and there are no visible on/off markings. A rocker switch on the front of the photometer which controls the current to the lamp may be confused with the mains power control switch as it is not labelled and there are no visible on/off markings. This is doubly confusing as an identical switch on the printer unit has a contrary movement.

An interesting safety device prevents the removal of fuses when the main input sockets were in but the current ratings of the fuses are not marked on either module (para 147). Owing to a possible printing error in the manual, the instructions for changing the voltage selection chip to alter the voltage are not clear.

The results of four safety checks carried out in accordance with the Safety Code are presented in Table 7 and are within acceptable limits. The effect of mains voltage fluctuation on the photometer and printer was investigated by testing the instrument at ± 10% of the rated 240 V. The printer functioned satisfactorily at between 216 and 264 V. The photometer worked at 264 V, but not at 216 V. It did, however, function at 220 V which is the lowest level to which any British supply rated at 240 V is likely to drop.

### Costing

The capital cost of the photometer plus printer but without the flow cell in the UK was approximately £3,700 at the time of evaluation (Summer 1979). A diluter and incubation bath (either the manufacturer's or other suitable alternative) are essential and would have to be available.

The equipment is designed to be used in conjunction with Boehringer Diagnostic reagents, but there is no restriction to these chemicals, and indeed any methods and reagents within the restrictions of the wavelengths available could be used. Reagent costing therefore is dependent upon the source of material and types of assays required.

For the manual system the manpower costs will be relatively high, but will compare well with other manual systems because of similar throughput times. For these reasons it is envisaged that the instrument will be used mainly for 'stat' analyses and it should be remembered that where such analyses are executed 'out of normal working hours' labour costs are budgeted separately and are not affected by the methods of analysis.

### Conclusions

During the three months of the evaluation the equipment functioned satisfactorily with no breakdown problems, except on a few occasions when the printer gave the result in blue instead of red type for a non-linear result in the kinetic mode. The operators found the instrument easy to use, and although some were initially confused by the large array of keys, the programming sequences were generally well described in the handbook and were easy to follow. The pre-programmed operational check-out procedures for verifying valid operation of the system were particularly useful. The location of on/off switches is not satisfactory, but this has been dealt with in detail in the electrical evaluation section. It is possible to obtain sensible but incorrect readings when the cuvette chamber is not completely closed. The lid should be fitted with a micro switch so that no readout is obtained if it remains open.

The functional evaluation was generally satisfactory. The instrument appeared to be very stable electronically as shown by the low level of lamp drift, the temperature stability, the reproducibility of the timing mechanism and absorbance values on the same sample in the same cell. The difference in absorbance at certain wavelengths between the Chemcode and a spectrometer (Table1) is a phenomenon often observed when filter instruments with relatively large band widths are compared with those using gratings. When the light source has a discontinuous spectrum as in this case, the difference is assumed to be due to spurious radiation, lamp impurities etc. It is also assumed that the narrow band widths quoted in the photometer specifications are a result of the low band width emission of the light source and not the filters. It has been noted however, that the divergence at 365, 405 and 436 nm is non-linear, appearing to be highest at an absorbance of about 0.5 to 0.6. This is curious as it does not appear to effect the linearity of the filter response nor the linearity of the endpoint chemistry methods. The analytical evaluation was aimed to cover the instrument's three modes of analysis and the available wavelengths, using control sera and patient specimens to determine precision and correlation with other laboratory findings. This was largely achieved, although the fixed point kinetic mode was not evaluated in detail and no method was available which required the 436 nm filter.

Three chemistries were investigated in the endpoint mode, each correlated well with the comparison method and two gave acceptable precision (see Analytical Evaluation – End-point Mode). For urea the precision was not acceptable, the problem probably being due to the difficulty in dispensing a sample volume of 5 µl which is below the range of the BCL Clinicon 2075 diluter. In addition there was some evidence to suggest that falsely low values were obtained with specimens which were not fresh. Because of the importance of urea as a 'stat' analysis it is essential that this problem is solved. The use of a method based on the Berthelot reaction would be more reliable.

The results obtained in the KIN mode were within acceptable tolerances. Although the coefficient of variation obtained with commercial sera were large, these were all on preparations with low activity and the precision improved at higher activity which is usual. The only assay carried out in the fixed point kinetic mode, γ-glutamyl transferase, gave a result which was not significantly different from the comparison method, although presumably some specimens give a non-linear response. This poses a question as to whether there is a need for this mode of operation, because although the time of analysis is less the quality of results is in doubt.

In conclusion, the authors feel that the Chemcode has a place in the routine clinical chemistry laboratory dealing with 'stat' work and assays with a low workload. Although

Table 7. Results of safety checks

| Test | Para | Recommended result | Observed result | |
|---|---|---|---|---|
| | | | Photometer | Printer |
| Leakage current | 72 | Not greater than 0.75 mA | less than 0.1 mA (in both directions) | 0.35 mA (in both directions) |
| Earthing | 61 | Not greater than 0.1 ohm | 0.1 ohm | 0.1 ohm |
| Insulation resistance | 95 | Not less than 10 Mohm | Greater than 100 Mohm at 500V | Greater than 100 Mohm at 500V |
| Fusing factor | 39,146 | Not greater than 2 | 0.26 | 0.35 |

capital cost is higher than some of its competitors it has the advantage of carrying out kinetic analyses in the KIN mode with monitoring by the operator for linearity within limits specified and hence producing a result of higher quality.

ACKNOWLEDGEMENTS
The authors wish to thank Boehringer Corp. Ltd. for the opportunity to carry out this evaluation and for supplying many of the reagents, and especially Mr Michael Clegg for his help and cooperation.

REFERENCES
[1] Trinder, P., *Journal of Clincial Pathology*, 1969, **22**, 246
[2] Moorehead, W. R., and Biggs, H. G., *Clinical Chemistry*, 1974, **20**, 1458
[3] Stromme, J. H., and Eldjarn, L., *Scandinavian Journal of Clinical Laboratory Investigation*, 1974, **33**, 291
[4] Searcy, R. L., Simms, N. M., Foreman, J. A. and Bergquist, L. M., *Clinica Chimica Acta*, 1965, **12**, 170
[5] "Electrical Safety Code for Hospital Laboratory Equipment, 1977" HMSO, London.

### Monitoring an average temperature in an automatic density assembly

**M.E.B. Brown* and R.G. Lidzey**
*The Laboratory of the Government Chemist, Cornwall House, Stamford Street, London, SE1 9NQ, UK.*

In the continuous measurement of temperature in a temperature-dependent system it may be inconvenient or impossible to make the measurement at the site where the variation is most important. This may, for example, occur in a water jacketed temperature controlled arrangement where there is difficulty in insulating sensor leads or fitting a mercury in glass thermometer due to the proximity of other equipment. In such cases it may be acceptable to take the average value at two or more other local sites, and in the example quoted an average temperature derived from sensors located in the inlet and exit water streams may be just as satisfactory.

The determination of strength of alcohol solutions by density requires precise temperature control. In an automated assembly [1] using a commercially built densimeter, the Anton Paar DMA55 (available from Standon Redcroft, UK) it was found necessary to have a continuous display of the density cell temperature. A variation of 0.01°C will introduce an error of 1 in $10^5$ in the density or 0.01% v/v in the apparent alcohol strength.

Thermistors were fitted into limbs of the thermostated water supply external to the instrument for the reasons stated above. Although a small diameter thermometer pocket is fitted on the cell, a thermistor fitted here is found to give misleading temperature indications and the present arrangement is preferred. Densities are measured at a nominal temperature of 20°C and a digital display of the temperature is given as a positive or negative departure from this value with a sensitivity of $10^{-3}$°C and a range of ± 2°C.

**Present address: Plasma-Therm Ltd., 6 Station Rd., Penge, London SE20, UK.*

### Circuit details

The two thermistors are connected in series in a bridge circuit forming one leg of the bridge (Figure 1). The remaining legs contain fixed resistors of the same total value. A voltage reference stabilised by two zener diodes supplies the bridge. Output of the bridge is via the 5.6 volt zener diode selected for its low temperature coefficient. The two 50 kohm resistors between the bridge and the first operational amplifier reduce the effect that the changing impedance of the thermistors have on the gain of the amplifier. A 0.35 volt potential drop across the forward biased signal diode provides a voltage offset to the second operational amplifier. The voltage is adjustable by a 5 turn ten k ohm potentiometer. The gain of the second operational amplifier is adjustable so that a change of ± 1.999°C in the mean temperature is displayed as ± 1.999 on the panel meter. The components are selected for stability, the resistors are TR5 type, the preset 5 k ohm twenty turn potentiometer is a wire wound precision type by Bourns Trimpot (Hadford House, 17/27 High Street, Hounslow, Middlesex TW3 ITE UK) and the thermistors are YS1 precision type 44031. (Sasco Ltd, PO Box 2000, Crawley, Sussex, RH10 2RV UK). The operational amplifiers, OP07 by Precision Monolithics, supplied by Bourns address have an ultra low offset voltage and current drift. A mains operated power unit by Gresham Lion (Gresham House, Twickenham Road, Feltham, Middlesex TW13 6HA UK) supplies ± 15VDC. A convenient check on the circuit function can be made by switching out the thermistors and connecting in a resistance equal to the combined thermistor values at 20°C.

The unit was calibrated by immersing both thermistors in a bath and adjusting the gain of the second operational amplifier over the ±2°C range with the 5 kilohm potentiometer and then setting the panel meter display to 0.000 for 20°C with the offset control. The sensitivity is 0.001°C with a 24 hour drift not exceeding ±0.005°C. When both temperatures are within ± 0.5°C of 20°C, the maximum error due to the non-linear characteristics of the thermistors is 0.01°C.

REFERENCES
[1] Submitted for publication Journal of Automatic Chemistry.

